# Improvement of Toluene Selectivity via the Application of an Ethanol Oxidizing Catalytic Cell Upstream of a YSZ-Based Sensor for Air Monitoring Applications

**DOI:** 10.3390/s120404706

**Published:** 2012-04-11

**Authors:** Tomoaki Sato, Michael Breedon, Norio Miura

**Affiliations:** 1 Interdisciplinary Graduate School of Engineering Sciences, Kyushu University, Kasuga-shi, Fukuoka 816-8580, Japan; E-Mail: mr-t-satoh@mms.kyushu-u.ac.jp; 2 Japan Society for the Promotion of Science, Chiyoda-ku, Tokyo 102-8471, Japan; E-Mail: m.breedon@astec.kyushu-u.ac.jp; 3 Art, Science and Technology Center for Cooperative Research, Kyushu University, Kasuga-shi, Fukuoka 816-8580, Japan

**Keywords:** indoor air monitoring, toluene, YSZ, mixed potential, ethanol, ppb levels

## Abstract

The sensing characteristics of a yttria-stabilized zirconia (YSZ)-based sensor utilizing a NiO sensing-electrode (SE) towards toluene (C_7_H_8_) and interfering gases (C_3_H_6_, H_2_, CO, NO_2_ and C_2_H_5_OH) were evaluated with a view to selective C_7_H_8_ monitoring in indoor atmospheres. The fabricated YSZ-based sensor showed preferential responses toward 480 ppb C_2_H_5_OH, rather than the target 50 ppb C_7_H_8_ at an operational temperature of 450 °C under humid conditions (RH ≃ 32%). To overcome this limitation, the catalytic activity of Cr_2_O_3_, SnO_2_, Fe_2_O_3_ and NiO powders were evaluated for their selective ethanol oxidation ability. Among these oxides, SnO_2_ was found to selectively oxidize C_2_H_5_OH, thus improving C_7_H_8_ selectivity. An inline pre-catalytic cell loaded with SnO_2_ powder was installed upstream of the YSZ-based sensor utilizing NiO-SE, which enabled the following excellent abilities by selectively catalyzing common interfering gases; sensitive ppb level detection of C_7_H_8_ lower than the established Japanese Guideline value; low interferences from 50 ppb C_3_H_6_, 500 ppb H_2_, 100 ppb CO, 40 ppb NO_2_, as well as 480 ppb C_2_H_5_OH. These operational characteristics are all indicative that the developed sensor may be suitable for real-time C_7_H_8_ concentration monitoring in indoor environments.

## 1. Introduction

Since the wider awareness of the environmental and health concerns that volatile organic compounds (VOCs) pose, the development of high-performance VOCs sensors has been of great interest [1–6]. This is due to their disruptive nature in atmospheric chemistry as well as their hazardous effects on the human body. For example, the production of photochemical smog [7], and the direct negative effects to our health, which are cumulatively referred to as sick building syndrome, are both exacerbated by the presence of atmospheric VOCs [8]. However, the development of reliable VOCs sensors have been hampered by the extremely low target detection levels, which are often in the order of several parts per billion (ppb) [9,10]. Additionally, negative effects on sensing performance can be caused by many interfering gases, such as HCs, NOx and H_2_O, *etc.* in the sensing environment, which often exist at significantly higher ppm concentrations [11–13].

Recently, we reported that a mixed-potential type gas sensor, which consists of yttria-stabilized zirconia (YSZ) and a NiO sensing-electrode (SE), gave sensitive responses towards toluene, a typical and often representative VOC which exists in indoor atmospheres at ppb levels [14,15]. In addition, low negative interferences of C_3_H_6_, H_2_, CO and NO_2_ were observed, suggesting a high possibility for a selective VOC sensor. This sensor may be useful for real world VOC monitoring and indoor sensing applications, such as VOC detection in conjunction with heating, ventilation, and air conditioning (HVAC) control systems for the prevention of sick building syndrome. The sensing performance of the developed sensor must also be capable of selectively discriminating against unique indoor gases when monitoring VOC levels. Ethanol (C_2_H_5_OH) is perhaps the most common interfering gas in indoor environments because the concentration of ethanol temporarily spikes owing to its culinary use, alcoholic beverage consumption, disinfectant use, and due to its adoption as a general solvent in some cleaning products [16]. In this paper, the sensing characteristics towards toluene and high concentrations of ethanol were evaluated for a NiO/YSZ-based sensor, aiming at the selective detection of ppb levels of toluene for indoor sensing applications.

## 2. Experimental

A tubular YSZ (8 mol% Y_2_O_3_ doped ZrO_2_, Nikkato, Japan); 10 mm in length and 3 mm in diameter, was used as both the solid-electrolyte and mechanical support of the sensor. NiO powder (99.9%, Kishida Chemical, Japan) was thoroughly mixed with α-terpineol; and the resulting NiO paste, as well as a commercial Pt paste (TR-7601, Tanaka Kikinzoku, Japan) were respectively painted on the outer and inner surfaces of the YSZ tube. The painted YSZ tube was dried at 130 °C, and then calcined in air at 1,000 °C for 2 h, to form the NiO-SE and Pt-RE. Pt wires were wound on the electrodes, acting as current collectors.

**Figure 1.**
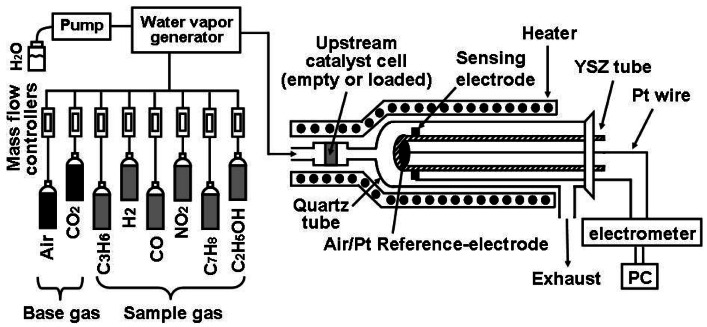
Schematic view of the gas sensor evaluation system.

The gas sensing evaluation system is presented in Figure 1. The system consisted of gas cylinders (NIST certified) equipped with mass flow controllers to accurately mix sample gas concentrations; a water vapor generator to humidify sample gas; and a digital electrometer which measures the potential difference between SE and RE as a sensing signal. A quartz cell loaded with 20 mg catalyst powder was applied upstream of the VOC sensor. The gas responses of the fabricated sensor were measured at an operational temperature of 450 °C, under the following conditions: 21 vol% O_2_, 1.35 vol% H_2_O (RH ≃ 32%) and 400 ppm CO_2_, in order to replicate a realistic atmospheric environment. The total gas flow rate was fixed at 100 cm^3^·min^−1^. The selected sample gases were 50 ppb toluene (C_7_H_8_) as a representative VOC gas, 50 ppb C_3_H_6_, 500 ppb H_2_, 100 ppb CO, 40 ppb NO_2_, and 80–480 ppb ethanol as interfering gases, considering the Japanese guideline value for toluene (70 ppb) [17]; and average or higher concentrations for interfering gases [11–13], to evaluate the sensor in challenging circumstances.

The gas-phase catalytic activity of Cr_2_O_3_, SnO_2_, Fe_2_O_3_ and NiO powders was evaluated under the same conditions as the constructed sensor. Each commercial oxide powder (Kojundo Chemical Lab. and Kishida Chemical, Japan) was sintered at 1,000 °C for 2 h, and 20 mg of each powder was separately loaded into a quartz catalytic cell maintained at 450 °C. The downstream gas concentrations of 50 ppb toluene and 80 ppb ethanol after passing through the catalytic cell was measured by a YSZ-based sensor utilizing a NiO(+20 wt% nano Al_2_O_3_)-SE, which was fabricated, as per our group's basic procedure for gas sensor fabrication [14,15].

## 3. Results and Discussion

**Figure 2.**
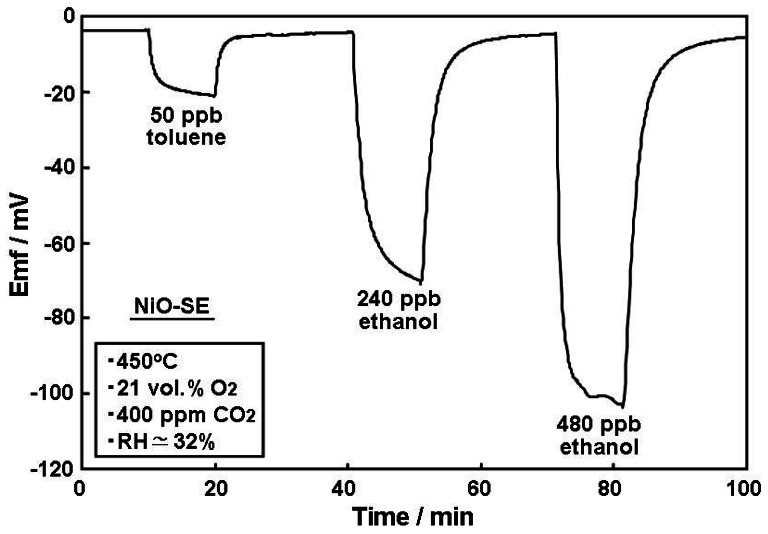
Response transients of YSZ-based sensor utilizing NiO-SE towards toluene and ethanol at an operational temperature of 450 °C under humid conditions (RH ≃ 32%).

As seen in our previous paper [15], a YSZ-based sensor utilizing NiO-SE gave high responses towards several kinds of VOCs, such as toluene, *m*-xylene, benzene, ethylbenzene, styrene, and formaldehyde; with low negative effects caused by C_3_H_6_, H_2_, CO and NO_2_. For the purpose of indoor sensor applications, the evaluation of sensing characteristics towards common indoor gases is of great interest. Figure 2 shows the response transients of the sensor using NiO-SE towards ppb levels of toluene and ethanol, with the later being one of the most significant interfering gases in indoor atmospheres, due to its higher average concentrations [16]. Unfortunately, the fabricated sensor exhibited preferential responses towards ethanol, rather than to the desired toluene. This behavior is similar to other potentiometric YSZ-based sensors reported elsewhere [1], indicating that NiO-SE has an extremely high catalytic activity toward the electrochemical reaction of ethanol rather than toluene, at the triple phase boundary (TPB). The electromotive force (emf) drift after 9 min exposure to either toluene or ethanol was less than −1 mV/min, indicating that for practical purposes the sensor had almost reached a steady state emf.

To decrease ethanol sensitivity and improve toluene selectivity, a gas-phase catalyst was applied upstream of the sensor to achieve selective ethanol oxidation. The application of catalysts for YSZ-based sensors has been reported for the selective detection of HCs [18], NOx [19] and NH_3_ [20], which dealt with high concentrations of gases at parts per million (ppm) levels exhausted from vehicles, where conditions are completely different from those found in indoor environments (several tens of ppb). Recently, we have reported that the lamination of a ZnO layer onto the SnO_2_-SE of a YSZ-based amperometric sensor can improve C_3_H_6_ selectivity to ppb levels in atmospheric environments [21].

To find a suitable catalyst for the selective oxidation of ethanol, the catalytic activity of four different oxides were evaluated by measuring the downstream concentrations of toluene and ethanol after passing through a catalytic cell loaded with one of the respective oxide powder. The downstream gas concentration was analyzed with a YSZ-based sensor utilizing NiO(+20 wt% nano Al_2_O_3_)-SE, which was confirmed to be sufficiently sensitive for the detection of ppb toluene and ethanol concentrations.

**Figure 3.**
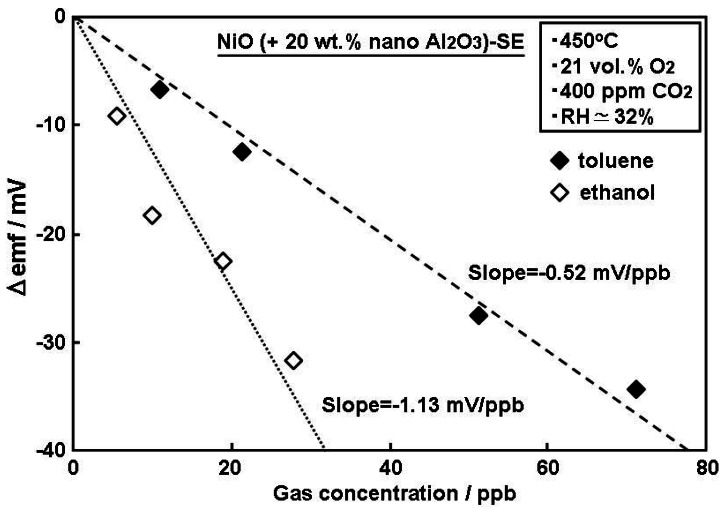
Calibration curves of YSZ-based sensor utilizing NiO(+Al_2_O_3_)-SE towards toluene and ethanol at an operational temperature of 450 °C under humid conditions (RH ≃ 32%).

Prior to evaluation, the calibration curves towards each sample gas were measured for the present sensor, as can be seen in Figure 3. The results given in Figure 3 indicated that the sensor showed almost linear trends of concentration dependence on sensitivity, indicating the approximate concentration determined by the sensor. The observed trend of the dependence was different from that of general mixed-potential type sensors [22–25] whose sensitivity typically varies linearly with the logarithm of gas concentration. This unusual linear behavior in the limited sensitivity region in Figure 3 was reported to be due to gas-diffusion limiting behavior in SE layer [26]. The slope of each calibration curve was −0.53 mV/ppb for toluene and −1.13 mV/ppb for ethanol. In Figure 3, gas sensitivity (Δemf) was defined as the difference in electromotive force (emf) measured in base gas and sample gas.

**Figure 4.**
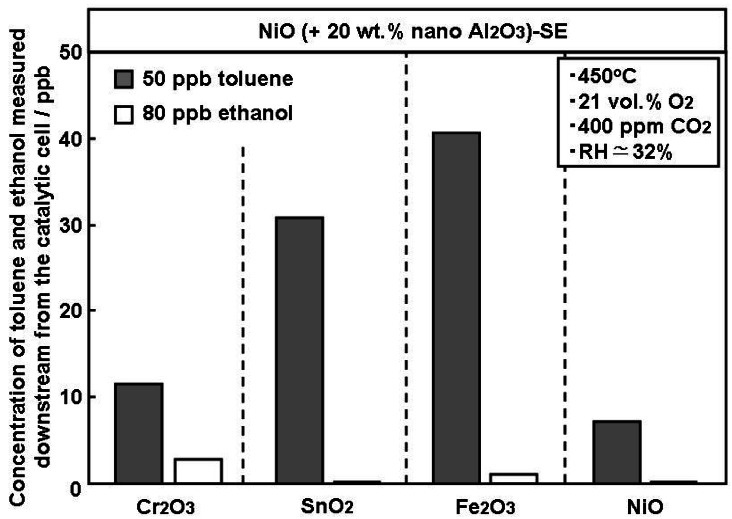
Catalytic activity comparison of different oxides for the oxidation of toluene and ethanol, evaluated with a YSZ-based sensor utilizing NiO(+Al_2_O_3_)-SE at an operational temperature of 450 °C under humid conditions (RH ≃ 32%).

Figure 4 compares the catalytic activity of various oxide powders for toluene and ethanol oxidation. The evaluation was performed under a humid and carbonized atmosphere at 450 °C, which is the same operational parameters as the YSZ-based sensor, considering the future potential for catalyst lamination onto the SE as a prospective research avenue. The downstream concentrations estimated by the YSZ-based sensor utilizing NiO(+Al_2_O_3_)-SE revealed that all catalysts have a higher catalytic activity for ethanol oxidation rather than toluene, which is expected as ethanol is generally easily adsorbed and catalytically decomposed on oxide surfaces, even at temperatures below 300 °C [27–29]. However, Cr_2_O_3_ and NiO also decomposed approximately 80% of toluene, indicating that the application of these catalysts in a sensing system would most likely cause a drastic decrease in toluene sensitivity. This result supports data presented in our previous paper [15], regarding the observation that an increase in the thickness of NiO-SE decreases toluene sensitivity. Among the oxides tested, SnO_2_ was selected as a suitable catalyst for the selective toluene sensing-system owing to its ability to selectively oxidize ethanol, while maintaining C_7_H_8_ response. Similary, Fe_2_O_3_ was also found to be effective at oxidizing C_2_H_5_OH, however as Fe_2_O_3_ incompletely oxidized C_2_H_5_OH, its use was discontinued for further experimentation. The ability of SnO_2_ to almost completely oxidize C_2_H_5_OH is of critical importance as there is often high ethanol concentration in indoor atmosphere (1.48 ppm) [16].

**Figure 5.**
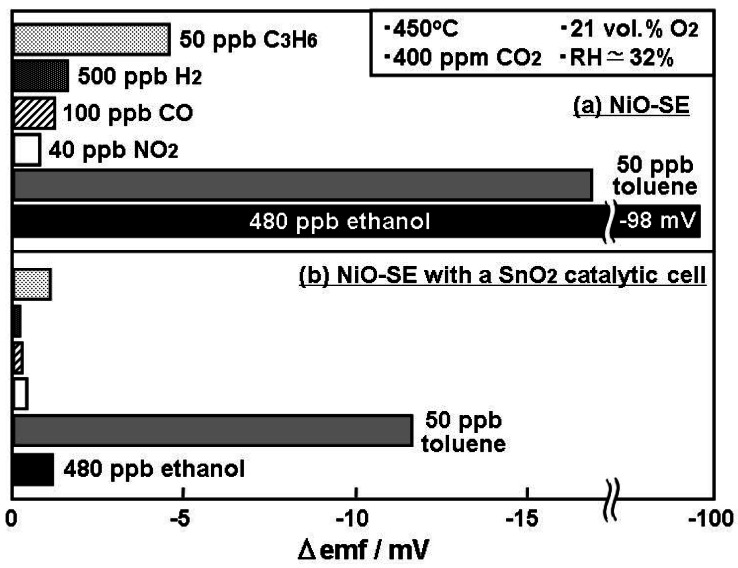
Comparison of cross sensitivities towards different gases (**a**) YSZ-based sensor utilizing NiO-SE; (**b**) YSZ-based sensor utilizing NiO-SE with SnO_2_ oxidation cell at an operational temperature of 450 °C under humid conditions (RH ≃ 32%).

A selective toluene sensing-system was constructed by placing a quartz cell loaded with 20 mg of SnO_2_ powder, upstream of the YSZ-based sensor utilizing NiO-SE. Figure 5 shows the comparison of cross sensitivities towards toluene and interfering gases, including high concentration ethanol for the sensor using NiO-SE with and without a SnO_2_ catalytic cell, at an operational temperature of 450 °C under humid conditions. It can be clearly seen that the application of the catalytic cell caused a drastic decrease in ethanol sensitivity; from −98 mV to −1.5 mV for 480 ppb ethanol by oxidizing ethanol in the SnO_2_ catalyst cell, although toluene sensitivity was also slightly affected. The sensitivities towards other interfering gases also decreased, which indicates that the SnO_2_ powder presumably partially catalyses C_3_H_6_, H_2_, CO and NO_2_, causing high toluene selectivity. The developed sensing system was confirmed to selectively detect very low concentrations of toluene at ppb levels, by catalyzing interfering gases. The investigation of this catalyst via the direct lamination of a SnO_2_ layer onto NiO-SE for a compact sensor is currently under investigation.

## 4. Conclusions

The application of a SnO_2_ catalytic cell upstream of a YSZ-based sensor utilizing NiO-SE resulted in a great improvement of toluene selectivity due to the oxidation of high concentration ethanol (480 ppb) before it reaches to TPB. The detectable levels of toluene (50 ppb) in the developed sensing system was found to be less than the indoor guideline concentration (70 ppb), established by the Japanese government for the prevention of sick building syndrome. In addition, effects caused by other interfering gases such as C_3_H_6_, H_2_, CO and NO_2_ were negligible, demonstrating that the developed sensing system could be utilized as a selective toluene-monitoring device capable of detecting ppb levels in real indoor environments.
